# Artificial Intelligence, Augmented Reality, and Virtual Reality Advances and Applications in Interventional Radiology

**DOI:** 10.3390/diagnostics13050892

**Published:** 2023-02-27

**Authors:** Elizabeth von Ende, Sean Ryan, Matthew A. Crain, Mina S. Makary

**Affiliations:** Division of Vascular and Interventional Radiology, Department of Radiology, The Ohio State University Wexner Medical Center, Columbus, OH 43210, USA

**Keywords:** interventional radiology, artificial intelligence, machine learning, deep learning, radiogenomics

## Abstract

Artificial intelligence (AI) uses computer algorithms to process and interpret data as well as perform tasks, while continuously redefining itself. Machine learning, a subset of AI, is based on reverse training in which evaluation and extraction of data occur from exposure to labeled examples. AI is capable of using neural networks to extract more complex, high-level data, even from unlabeled data sets, and better emulate, or even exceed, the human brain. Advances in AI have and will continue to revolutionize medicine, especially the field of radiology. Compared to the field of interventional radiology, AI innovations in the field of diagnostic radiology are more widely understood and used, although still with significant potential and growth on the horizon. Additionally, AI is closely related and often incorporated into the technology and programming of augmented reality, virtual reality, and radiogenomic innovations which have the potential to enhance the efficiency and accuracy of radiological diagnoses and treatment planning. There are many barriers that limit the applications of artificial intelligence applications into the clinical practice and dynamic procedures of interventional radiology. Despite these barriers to implementation, artificial intelligence in IR continues to advance and the continued development of machine learning and deep learning places interventional radiology in a unique position for exponential growth. This review describes the current and possible future applications of artificial intelligence, radiogenomics, and augmented and virtual reality in interventional radiology while also describing the challenges and limitations that must be addressed before these applications can be fully implemented into common clinical practice.

## 1. Introduction

Artificial intelligence (AI) is the development of computer algorithms to process and interpret data as well as perform tasks with partial or complete autonomy, while continuously refining its logic and decision making. Only with the more recent development of powerful computational hardware capable of collecting, storing, and processing large amounts of data has the field of AI become relevant to radiology. Specifically, the field of interventional radiology (IR) is in a unique position to benefit from advances in AI to not only improve image processing, but also guide and predict outcomes of their minimally invasive procedures.

**Figure 1:**
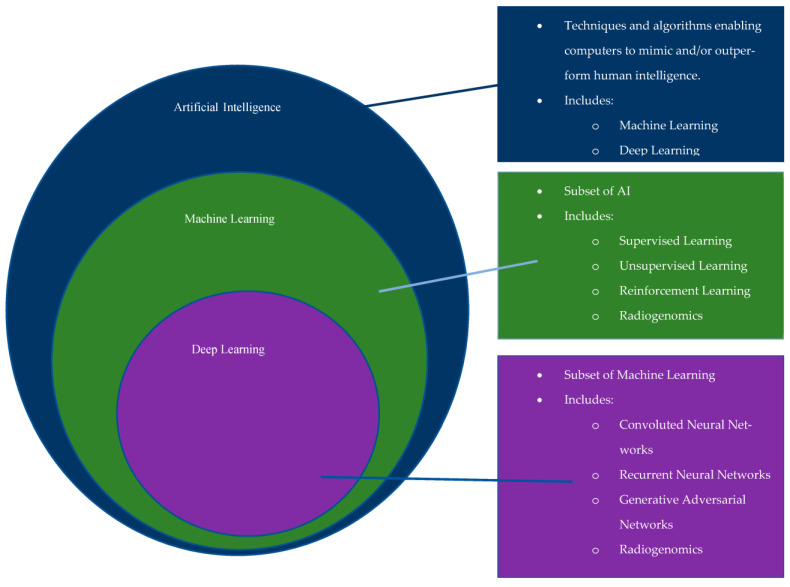
Schematic outlining the relationship between AI, ML, and DL, as well as aspects that make up each of them individually.

First officially introduced in the 1950′s, the growth of AI began with the introduction of artificial neural networks (ANN), an idea inspired by biologic neural networks in which the passage of information occurs via inputs and outputs from adjacent neurons. Since its introduction, there has been further progression into computational learning models, which include machine learning (ML) and deep learning (DL) (Figure 1) [1]. ML is based upon “reverse training”, in which its education occurs through exposure to specific, labeled data [1,2]. DL is a specialized subset of machine learning built from multilayered artificial neural networks (ANN) for use in more complex, higher-level tasks [1,2]. An ANN is a computational model that includes multiple levels of learning algorithms, or input and output ‘neurons’, and if one of these layers involves a convolutional filter, then it is classified as a convolutional neural network (CNN).

With the incorporation of neural networks, DL can automatically discern information from large sets of unlabeled data by training a CNN with numerous neural layers, between input and output, that contribute to the plasticity of the DL [1]. This allows DL to better emulate human intelligence, reasoning, and learning [1]. These algorithms can even identify specific characteristics of pathology that are beyond human discernibility. However, because of the need to train its neural networks, DL currently has limited applications in fields like interventional radiology where the case data is limited and often highly variable.

A constraint of AI’s current dependence on neural networks is the reliance on data-rich domains to train algorithms. The field of diagnostic radiology is optimal for such training as it is a unique data-rich specialty that has progressed rapidly in the modern age of technology. AI has already been successfully implemented in several areas of diagnostic radiology which has been shown to improve efficiency and patient outcomes when used in conjunction with trained radiologists. AI has already been successfully used to assess brain perfusion in acute strokes, delineate brain tumors, and protocol radiological studies. Although many of these listed examples are considered diagnostic radiology specific, there can be substantial overlap between diagnostic radiology and interventional radiology. The rapidly growing field of research called “Radiogenomics,” a close relative of AI, combines ML and DL image processing with clinical, histological, and pathological data, in an attempt to correlate precise imaging patterns with pathologic and/or histologic subtypes [3]. Though centered around its ability to extract complex data from medical images, the data obtained will help tailor patient-specific IR treatments. The applications for AI in IR continue to increase with advancing modern technology and the evolving healthcare landscape.

AI has the ability to further revolutionize healthcare, specifically for IR, through precision diagnosis, customized treatment plans, and real-time procedural support. Furthermore, although not a direct form of AI, similar fields such as augmented reality (AR) and virtual reality (VR) stand to improve physician education and training, improve patient understanding, and enhance procedural guidance as well as reduce risk and procedural complications.

The purpose of this review is to highlight the evolving applications of AI in IR, utilizing the previously described techniques, in the pre-procedural, intra-procedural, and post-procedural settings to improve patient selection, treatment planning and execution, procedural training, intraprocedural augmentation, and treatment follow-up. While AI has many uses, the complexity of the pre-procedural, intra-procedural, and post-procedural applications of AI in IR has presented several challenges and ethical dilemmas that have limited its integration when compared to fields like diagnostic radiology. We further explore current limitations to the progress of AI in IR, and ethical considerations that arise during the adoption of these nascent technologies. As AI has the potential to become more integral to the everyday workflow of both diagnostic and interventional radiologists, it is crucial to understand its various applications and limitations.

## 2. AI Applications

Applications of AI in IR can be divided into pre-procedural, intra-procedural, and post-procedural categories, as summarized in Table 1. Pre-procedural applications include, but are not limited to, patient selection as well as the utility of radiogenomics, AR, and VR. Intra-procedural applications include, but are not limited to, procedural guidance and radiation exposure. Post-procedural applications are tailored to the evaluation of procedural outcomes and follow-up.

### 2.1. Pre-Procedural Applications

#### 2.1.1. Patient Selection

Patient selection is crucial for a treatment’s effectiveness, and therefore, the ability to determine which therapies will be most effective for which patients is essential. A multidisciplinary approach to treatment is a key facet of IR, as numerous treatments are decided after multidisciplinary conferences and tumor board discussions, as well as in-depth risk-benefit reviews. AI models have the potential to aid in optimal patient selection by impartially assessing risk and predicting the potential outcomes of therapy [11]. A reliable method for predicting the benefit of treatment prior to its completion would be a significant advancement in the field. For example, Morshid et al. (2019) created an algorithm to predict the response of hepatocellular carcinoma (HCC) following transcatheter arterial chemoembolization which outperformed traditional systems [4]. Similarly, Daye et al. (2019) demonstrated the use of ML in the evaluation of pre-ablation CT texture patterns to predict post-treatment local progression following tumor ablation for adrenal metastases with an accuracy of approximately 95% [1,12]. By predicting which patients will have better responses to different treatments, interventionalists will be able to protect patients from the adverse effects of ultimately ineffective treatments and efficiently delegate limited treatment resources to patients with a greater likelihood of response.

Furthermore, the creation of algorithms to produce a summarized report of pertinent patient-specific information would not only be more efficient in daily practice but would also likely reduce human errors [1]. Incorporation would assist providers in making the most thorough and accurate therapeutic decisions for their patients [1,5]. Similar algorithms have been proposed for safety screening, a useful tool for example in pre-procedural analysis prior to MRI-guided procedures or in patients with contrast allergies [1].

#### 2.1.2. Radiogenomics

**Figure 2:**
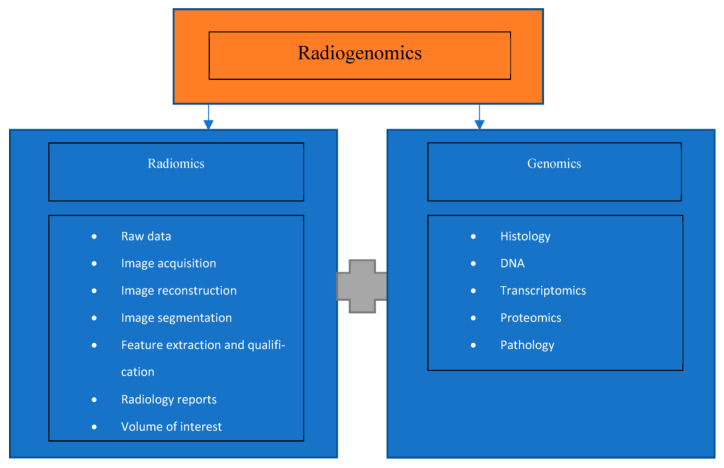
Schematic diagram outlining the aspects of radiogenomics.

The emerging field of radiogenomics combines medical imaging and molecular pathology, as shown in Figure 2, to improve diagnosis, prognosis, and treatment outcomes [3]. There is a new realization that medical imaging contains a significant amount of “untapped” clinically relevant data that was not previously understood [3]. The ability to foresee an outcome or benefit of treatment prior to performing it is a major challenge in interventional radiology. However, the adoption of DL has the potential to mitigate this challenge [13]. If accurate diagnoses are possible without the need for tissue sampling, such is the case for HCC diagnosis on MRI, it would decrease unnecessary procedures, leading to decreased patient risk and a decrease in hospital cost [5].

Furthermore, the development of radiogenomics could be crucial to IR and its role in the treatment of oncology patients, such as those with HCC, renal cell carcinoma (RCC), colorectal cancer (CRC) with metastases to the liver, and lung cancer patients [3]. For example, radiogenomic studies have demonstrated potential in the correlation of HCC gene patterns with aggressive imaging features on CT, such as infiltration or microvascular invasion. As these are indicative of a poor prognosis, it would be crucial to detect, or, at a minimum, suggest these findings on initial imaging in order for the proper treatment option to be chosen as quickly as possible [3]. Additionally, radiogenomic studies have been performed on RCC indicating associations between CT imaging features with tumor mutations and therefore clinical outcome [3]. As there is evidence demonstrating loss of certain mutations with increased aggressiveness of RCC tumors and worse survival rates, radiogenomic-based triage tools would be helpful for determining whether RCC patients would benefit most from surgery or IR intervention with thermal ablation [3].

Overall, these applications have the potential to more accurately prognosticate and predict a patient’s response to a particular treatment, creating a more tailored and specific treatment approach. These augment the role of interventional radiologists as clinicians who partake in the treatment plan of patients, rather than strictly proceduralists. It is important as clinicians to establish accurate prognoses, as early as possible, and determine which patients would benefit most from specific treatments while decreasing patient risk, radiation exposure, and hospital cost as much as possible.

#### 2.1.3. Augmented Reality and Virtual Reality

Advances in AR allow operating physicians the ability to visualize procedures and determine their desired approach in the pre-procedural setting via 3D image rendering and manipulation [1]. Incorporation into clinical practice would enable visualization of difficult anatomy and/or improved procedural technique without added risk to patients [1,14]. For example, the degree of atherosclerotic plaque and its potential effect on wires and catheters can be determined preoperatively rather than intraoperatively [1]. This may not only improve the efficiency and performance of a procedure, but potentially decreases radiation to the patient and operator.

Furthermore, development of VR simulations could allow patients a pre-procedural virtual experience of the procedure. Although this would only be speculative, these simulations may improve a patient’s understanding of their procedure, thereby improving informed consent.

### 2.2. Intra-Procedural Applications

#### 2.2.1. Procedural Guidance and Support

AI has the potential to assist in and improve procedures in a variety of methods integral to IR, such as image fusion, catheter positioning and probe trajectory, vessel analysis or information regarding the availability of angiography suite supplies [1,2,6,11]. The most developed intraprocedural applications of DL techniques to date have been in the synthesis of pre-procedural 3D anatomic data fused onto 2D real-time fluoroscopic images for improved guidance during procedures. The ability to fuse pre-procedural images onto 2D fluoroscopic images allows for real-time feedback and thus advanced precision during biopsies and ablations. For this process, matching artificial intelligence software is incorporated into virtual and augmented reality to perform automatic landmark recognition through fiducial markers and motion compensation [1]. More recently, this technique has been applied to vascular procedures, such as the angiographic localization of a bleed [1]. Additionally, DL methods being studied for use in tumor ablation therapy include optimization of probe trajectory and selection of energy settings to maximize tumor treatment while simultaneously minimizing injury to adjacent tissue [1,6].

Another area of potential improvement for IR procedures includes the generation of digital subtraction angiography (DSA), a method of subtracting a mask image from the real-time angiogram. This technique requires patient cooperation as patient motion causes misregistration artifacts. DL algorithms utilizing generative adversarial networks for the creation of DSA images from a single live image without mask data acquisition, such as those suggested by Gao et al. (2019) would circumvent these issues of artifact [11,15].

Due to the numerous vascular interventions performed by IR providers, vessel analysis is also an optimal area for AI development. A presentation by Molony et al. at the Transcatheter Cardiovascular Therapeutics 2018 annual meeting demonstrated the ability of ML and IVUS to perform vessel analysis in cardiology procedures [2,16]. The use of IVUS in interventional radiology procedures is not a novel idea, and therefore these ML methods would be easily transferable to vascular analysis and post-treatment evaluation for IR procedures [2,16]. Vessel analysis with AI has similarly been studied by Cho et al. (2019) through the development of an AI algorithm capable of estimating real-time fractional flow reserve in coronary angiography, a process also easily transferable to IR procedures for peripheral arterial disease [1,17].

Lastly, AI may demonstrate procedural support by providing information regarding supply stock availability [5]. Currently, this information is amassed either beforehand or by other team members, which is not only time consuming but also introduces unnecessary errors. However, the introduction of touchless devices such as eye-tracking systems or voice-driven smart assistants in the IR suite could alleviate some of these issues [1]. Furthermore, voice recognition and gesture-capture camera systems have been studied for various actions such as turning on and off operating room machinery or operating technology while in the IR suite [5,18]. This would reduce the time and personnel needed to perform these tasks. Augmented reality embedded in lead glasses has also been evaluated to display important information to the operator while scrubbed into cases [5,19,20]. These have also been evaluated as smart assistants to help make suggestions intraoperatively on things like sheath size and deployment of different stents which may be time saving but also advantageous to the novice provider [5]. Additionally, the use of smart assistants could be beneficial for cost analysis, as a greater knowledge of device costs could lead to more cost-effective decision making intra-operatively [5,21].

#### 2.2.2. Radiation Exposure

Intraprocedural radiation exposure has the potential for substantial reduction with the utility of AI. For example, it has already been evaluated in endoscopy with AI-equipped fluoroscopy that reduces radiation exposure by 38% via ultrafast collimation [7,11]. The incorporation of AR, such as multi-modality image fusion via superimposition of pre-procedural 3D anatomic data onto 2D fluoroscopic images for improved guidance, as well as the use of adversarial networks for the creation of DSA images without the acquisition of mask images, would each individually and cumulatively decrease the necessary images obtained during a procedure and therefore the amount of radiation to the patient [1]. Furthermore, AI algorithms by Zimmermann et al. (2020) utilizing mobile eye-tracking glasses determined the amount of avoidable radiation per procedure was approximately 11 min. This is the amount of time the x-ray was on while the operator was not looking at the fluoroscopy screen [8,22]. Similarly, Bang et al. (2020) demonstrated significantly lower radiation to both the patient and operating personnel with the use of AI enabled fluoroscopy systems vs. traditional systems [7,22]. These applications are crucial to both patients and operators. Many of the patients in IR undergo frequent procedures for maintenance, such as routine nephrostomy or biliary drain exchanges and routine fistulography and intervention for patients with dialysis access. Therefore, even a small decrease in radiation for each procedure will generate an even larger cumulative decrease over time (7, 8). Likewise, interventional radiologists and technologists perform numerous procedures on a daily basis, and therefore small decreases in radiation for each procedure produces a much larger cumulative decrease in their total lifetime radiation exposure (7, 8).

### 2.3. Post-Procedural Applications

#### Treatment Evaluation and Follow-Up

Rapid, accurate, and objective assessment of the outcomes of IR procedures is critical. Having a clear understanding of these post-procedural outcomes will improve treatment predictions and future clinical decisions [11]. These outcomes can further be compiled into longitudinal studies that depend on systematic, objective, and reliable assessments throughout the research program. Finally, to make the results of the longitudinal studies generalizable, standardized objective outcome measures are necessary for multi-site clinical treatment research programs.

While diagnostic radiology studies have demonstrated the utility of AI to improve the accuracy, objectivity, and timing of imaging analyses, there have been limited published applications on IR post-procedural outcomes research [11]. An example of where AI applications have been used successfully in IR involves the use of a decision tree, more specifically a Random Forest, in which relationships can be made from complex data sets [23]. This has been used successfully in IR to predict pneumothorax following CT-guided lung biopsy, in-hospital mortality following transjugular intrahepatic portosystemic shunt, and length of hospital stay following uterine artery embolization [23]. These applications were possible due to the availability of large volumes of patient-specific demographics and clinical data in the electronic health records [23]. Based on these applications, it would therefore be feasible for similar methods to predict other relevant and actionable clinical outcomes, such as the development of acute kidney injury (AKI) following intraprocedural contrast usage [24].

Within the realm of interventional oncology, in order to develop a more valid and reliable assessment of Response Evaluation Criteria in Solid Tumors (RECIST) following chemotherapy, which depends on a reader’s measurement of tumor volume, Kidd et al. (2022) validated a fully automated Convolutional Neural Network (CNN) to calculate tumor size and treatment response [9]. This DL model could be applied more reliably and objectively to assess the outcomes of interventional radiology procedures, such as liver metastases, than traditional expert based RECIST. Comparably, Dohan et al. (2020) demonstrated the ability of AI to predict overall survival and identification of “good responders” more accurately than RECIST in the evaluation of colorectal liver metastases [10,11].

Similarly, in order to develop a more objective, standardized, and rapid assessment of mechanical thrombectomy outcomes in the treatment of acute ischemic stroke, Nielsen et al. (2021) designed a DL method to determine scores of thrombolysis in cerebral infarction (TICI) [25]. This artificial intelligence algorithm facilitates a more rapid, accurate, and reliable outcome, which can be used to develop more meaningful and effective management plans and prognoses as well as incorporate the findings into a larger longitudinal and multi-site research program. Likewise, Saillard et al. (2020) developed DL algorithms based on digitized histological slides to build models for predicting the survival of patients after hepatocellular carcinoma resection, a paradigm that can also be used following interventional radiology procedures, such as resections and ablations, to investigate the benefits of adjuvant systematic therapies [26].

These clinical studies illustrate the potential benefits of using AI to measure outcomes following IR procedures. Interventional oncology stands to benefit significantly as the growth of AI in post-procedural follow-up continues to allow for more specific and tailored treatment of oncology patients. Further research is clearly needed to apply the growing body of DL methods being developed for imaging analyses to assessments in IR post-procedural evaluation.

## 3. Training and Education

Advances in ML combined with VR simulation programs create new methods of teaching and preparation, allowing trainees the ability to practice procedural skills in a simulated environment [1]. Currently, there are already orthopedic surgical simulations being used in training, created from patient-specific anatomic modelling data from cross-sectional imaging and manual image segmentation [2,13]. Related simulations have been developed for IR education and training.

A unique aspect of IR training encompasses the development of spatial and cognitive awareness, tactile sensation and motor techniques that are required to operate IR equipment efficiently and successfully [27]. As the conventional training approach of “see one, do one, teach one” is replaced with “see many before doing many,” trainees have less hands-on experience than ever before [27]. The estimated 10,000 h of practice required to attain an experienced level of expertise becomes more difficult to accomplish in today’s training programs [27,28]. Inadequate proficiency leads to higher complication rates or operator errors, longer procedural times, and increased radiation to patients and operators [27,28].

Conversely, the implementation of VR simulation systems in education programs could counteract this predicament to provide trainees with sufficient hours of experience. Further, as case mix varies across institutions, IR physicians may possess very different skill sets based on their training environments [28]. Simulation databases could help expose trainees to a wider case variety. The incorporation of VR simulation systems coupled with standard teaching methods would ensure optimal training in a safe and effective environment, with the added benefit of reduced procedure times and operator errors [27,28].

ML and VR can improve IR education and procedural proficiency in both a national and international context. Through VR, more interventionalists can be trained in areas that have limited training programs, educators, and resources. The same data sets used to train the AI program could be utilized as education cases with standardized reports as the answer key. Assisting in the training of interventional radiologists worldwide would also serve to bolster the number of diverse cases and data sets. More diverse international cases would also ensure that the AI program does not become inherently biased to the anatomy and pathology of a single group of patients.

## 4. Limitations

In IR, there are different logistical and ethical obstacles that impede the implementation of AI into practice, as shown in Table 2. From a logistical perspective, the obstacles to AI implementation include small datasets relative to diagnostic radiology, standardization of AI learning, variations in patient anatomy and pathology, and difficulty incorporating and coordinating new technology into established healthcare systems [1].

A substantial number of standardized cases is required to build the foundation for an AI neural network. As IR is a relatively newer field of medicine, there are fewer established cases available to train the network. Establishing a sufficient repository of cases will require cooperation and data sharing between different healthcare systems, both nationally and potentially internationally [29]. That cooperation in itself is difficult to achieve given corporate competition and proprietary interests and could even act as a potential risk to violating patient privacy [30]. If cases are being contributed from different institutions, this will inevitably create inconsistencies in protocoling, procedure approach, reporting language, and subjective assessments of severity. Preventing inconsistencies requires standardization of practice across institutions and the establishment of a common lexicon [31]. Even if this was feasible, it would also require the creation of a central quality control agency to oversee this multifaceted project and ensure that this standardization was being upheld [30]. Currently, the regulation of AI in healthcare is subjective and poorly delineated across health systems and national governances [30,32].

Diagnostic radiology is a data-rich specialty whose progression in the modern age of technology has enabled it to combat AI’s neural networks’ reliance on data-rich domains to train their algorithms. However, in comparison to diagnostic radiology, interventional radiology is a relatively newer field with fewer total cases and a smaller network of physicians collecting new data. To overcome the limitation of attaining large quantities of high-quality data sets, interventional radiology could utilize techniques developed by neuroradiology researchers working to improve AI brain tumor delineation. These researchers have created data augmentation techniques that improve the generalization capabilities of deep neural networks by generating synthetic training examples. Data augmentation categories include elastic transformations, affine image transformations, pixel-level transformations, and various approaches for generating artificial data. A disadvantage of affine transformations in brain tumor AI training is that it can produce correlated images and generate anatomically incorrect examples [37]. Recent innovative research has also investigated building algorithms that generate artificial images, for example based on tumor growth models, that can be followed as a separate modality by other techniques to ensure the correctness of such phantom/artificial images given that they were found to still produce valid tumor characteristics [37]. If techniques similar to the Batch Adjusted Network Gradients (BANG) were modified for IR, they could allow for more representative and extensive training data as well as augmenting cases in real-time to improve the robustness of the deep learning program in previously imperfect examples [38].

Once the AI system is established, it may be difficult to ensure that it is performing optimally which could silently and detrimentally affect patient care. A complex multifaceted AI system that has unclear mechanical rationale and limits can be prone to debugging errors and requires frequent iterative feedback to ensure it is learning correctly [1]. Therefore, for AI to be applicable in radiology it not only needs to be able to process images correctly, but it also must have a separate functional self-monitoring system that ensures the quality of its results [1].

IR is rapidly evolving from a technological perspective, and it may be difficult to integrate AI systems into the constantly progressing equipment and software used for procedures and data analysis. For AI systems to provide benefits in a clinical/procedural setting, they must function seamlessly with both old and modern imaging scanners and software. A further complication of this integration is that, within a single health system, there are various technologies likely designed by different companies in different countries, each of which could potentially be incompatible with AI processing.

In addition to technological barriers to AI integration into clinical practices, hospital staff and their ability to adapt to new technology can also be a barrier. Both academic and private practice hospital staff come from a variety of backgrounds, and some may be limited by their ability to operate new AI technology or their desire to disrupt the current flow of their established clinical practice. Many physicians operate at a high level and may believe that new AI technology will only serve to disrupt their process or make errors that could harm their patients [11]. Private practices especially may be more likely to distrust AI software as it could detrimentally affect their immediate productivity and compensation. Furthermore, many IR private practices do not perform the extremely complex vascular procedures that AI and augmented reality have the highest potential to improve, which makes the technology less desirable. Like with any new technology, implementing AI tools into an established practice is an inherently time and resource consuming process that may risk being poorly received by staff.

The intraprocedural implementation of AI into IR has its own set of limitations. During procedures, interventional radiologists use their finely-honed technical expertise along with split second decision making to ensure success. As with all technology, there is a risk of technical difficulties or system failure that causes the program to freeze or severely delays output functions. If an AI program cannot keep up with physicians, then it cannot be relied upon which will drastically reduce its procedural applicability [33]. At the end of the day, it is the interventional radiologist who will need to make decisions based on the input from the AI system and make patient care decisions [35].

Furthermore, there is wide variety in normal patient anatomy. The ability of AI to distinguish the variations in normal vs. pathological could be a major challenge. Differences in size, ethnicity, gender, age, and congenital anomalies can greatly alter the landscape of a patient for interventional procedures and imaging. The ability for AI programs to tolerate this variability is unknown.

A significant barrier to the implementation of VR simulation systems is the significant cost of such a large technological investment. Although a reasonable concern, future studies may suggest overall cost-saving [27]. The cost of simulation devices, although substantial, could more substantially reduce the cost of procedural complications and prolonged hospital stays from inexperienced operator complications. Although slightly different, simulation-based central venous catheter (CVC) courses significantly reduced CVC-related infections and hospital costs [27]. Moreover, considering the financial burden and potential patient benefit, it may be beneficial for training programs to share the financial burden with other departments, such as cardiology and vascular surgery [39].

Overall, at this time, there are significant advancements being made in AI applications in the medical setting, particularly in diagnostic radiology. While there are many limitations and fewer widely implemented AI applications currently in use in the field of interventional radiology, there are many prospective applications that will develop as technology progresses and a greater understanding of AI is achieved.

## 5. Ethical Considerations

Beyond the technological, economical, and biological obstacles to AI implementation in interventional radiology, there are also ethical dilemmas to consider. For example, when considering the variability of patient anatomy across the globe, no AI program data set can be adequately trained for all variations of both normal anatomy and overlapping disease presentations. Inadequate training and poor differentiation can lead to misdiagnosis and procedural complications when using AI [34]. The prevalence of different diseases as well as pretest and posttest probabilities also vary between patient populations. With different populations and pathologies running the risk of being underrepresented in the reference data sets, this creates a risk of a breach of justice in medical ethics. For AI to be used in a responsible and ethical manner, there needs to be a coordinated effort to prioritize human rights and freedoms, including privacy, dignity, and safety [35]. Radiologists and AI system programmers will need to altruistically advocate for patient care and ignore monetary influences.

Other ethical considerations include patient privacy, patient safety, and the responsibility of physicians utilizing AI assistance [35]. To efficiently amass enough standardized cases to train the AI program, it will be crucial for healthcare systems to share patient information since no single center will see sufficient case volume and variety. Patients will have to provide informed consent to share their private medical records with medical and corporate entities, and these groups will have to ensure that this information is protected and not misused [35].

When seeking to improve the efficiency of radiology services, it is important to prioritize patient safety over procedural and diagnostic interpretive speed. Physicians serve as advocates for their patients and thus must enact high standards for AI programs to protect patients from adverse consequences. Interventional radiologists must oversee and intervene if AI assisted procedures or imaging study reads are causing errors such as missing ischemic strokes or incorrectly mapping vessels. As part of patient safety, physicians are directly responsible for the proper management of their patients [36]. However, this relationship could be muddled by the introduction of AI. If AI programs are allowed to automate patient scheduling, image analysis, and post-procedural follow-up and prognosis, then the accountability of the associated radiologist becomes unclear [36].

## 6. Conclusions

Numerous, impactful areas of IR stand to benefit greatly from the incorporation of AI. Integration of these techniques would not only benefit procedural planning and performance as well as treatment follow-up, it is also poised to improve patient experience, decrease radiation exposure to both the patient and operators, and potentially decrease hospital costs and adverse events. The benefits of AI in IR are far-reaching and can help on an individual patient level by improving scheduling and the efficacy of minimally invasive procedures, but also on an international level by optimizing global radiology education. Multiple studies have already demonstrated the positive impact of AI integration in the IR setting, and the capabilities are only getting broader with advancing medical imaging technology and more comprehensive prognostic models. There are definite limitations that must be overcome and ethical considerations which must be taken into consideration before the wide breadth of AI applications is demonstrated in daily practice. However, continued enthusiasm as well as research and data collection are key to unlocking the potential AI applications in IR.

## Figures and Tables

**Table 1:** Summary of pre-procedural, intra-procedural and post-procedural AI applications with associated examples and references.

AI Applications		Example	Reference
Pre-Procedural Applications			
	Safety-screening	Algorithms useful in prescreening of patient charts	Gurgitano et al. [1]
	Patient Selection	Patient selection using ML- and DL-based predictive models to categorize patients as responders and non-responders.	
		Pre-procedural virtual experience of their upcoming procedure	
	Augmented Reality	Visualization of difficult anatomy	
	Virtual Reality	New method for teaching and training	
	Radiogenomics	Combining ML and DL image algorithms with molecular pathology to improve preprocedural diagnosis, prognosis and outcome.	Moussa et al. [3]
	Patient Selection	Algorithms designed to predict the response of HCC to TACE prior to the procedure	Morshid et al. [4]
Intra-procedural Applications			
	Image Fusion	Fusion of 3D anatomic data onto 2D fluoroscopic images for advanced precision during biopsies/ablations and for angiographic localization of bleeding	Gurgitano et al. [1]
	Smart-Assistant Devices	Augmented reality embedded lead glasses capable of displaying useful/relevant information to the operator while he/she is scrubbed into a case	Iezzi et al. [5]
		Voice-recognition and gesture-capture camera systems for operating IR suite machinery.	
	Cost Effectiveness	Smart assistance capable of analyzing device cost prior to use	
	Ablation Probe Trajectory	DL algorithms for optimization of probe trajectory in tumor ablations to maximize tumor treatment while minimizing injury to adjacent structures	D’Amore et al. [6]
	Radiation Exposure	Decreased radiation using AI enabled fluoroscopy systems	Bang et al. [7]
	Radiation Exposure	Mobile eye-tracking glasses for estimation of avoidable radiation per procedure	Zimmermann et al. [8]
Post-procedural Applications			
	Treatment Follow-Up	Fully automated CNN to calculate tumor size and treatment response	Kidd et al. [9]
	Treatment Follow-Up	Algorithms designed to predict overall survival as well as categorization of “good responders” and “bad responders” following treatment	Dohan et al. [10]

**Table 2:** Limitations and ethical considerations associated with the implementation of AI into IR.

Limitations/Challenges
Ensuring optimal AI learning [1]
Small datasets for AI training [29,30]
Standardization of IR practice [31,32]
Procedural applicability and incorporation of new technology into an established healthcare system [33]
Variations in patient anatomy and pathology [34]
Currently there are fewer suitable uses for AI in IR compared to diagnostic radiology [11]
**Ethical Considerations**
Conflicts of interest between AI developers and radiologists [8]
Effort to prioritize human rights and freedoms such as privacy, dignity and safety [35]
Disruption of the direct responsibility between physicians and their patients [36]

## Data Availability

Not applicable.
